# Hemorrhagic lesion with detection of infected endothelial cells in human bornavirus encephalitis

**DOI:** 10.1007/s00401-022-02442-3

**Published:** 2022-06-03

**Authors:** Friederike Liesche-Starnecker, Martina Schifferer, Jürgen Schlegel, Yannik Vollmuth, Dennis Rubbenstroth, Claire Delbridge, Jens Gempt, Stefan Lorenzl, Lea Schnurbus, Thomas Misgeld, Marco Rosati, Martin Beer, Kaspar Matiasek, Silke Wunderlich, Tom Finck

**Affiliations:** 1 Department of Neuropathology, School of Medicine, Institute of Pathology, Technical University of Munich, Munich, Germany; 2 Medical Faculty, Institute of Pathology and Molecular Diagnostics, University of Augsburg, Stenglinstraße 2, 86156 Augsburg, Germany; 3 Institute of Neuronal Cell Biology and Munich Cluster of Systems Neurology (SyNergy), Technical University of Munich, Munich, Germany; 4 German Center for Neurodegenerative Diseases, Munich, Germany; 5 Institute of Diagnostic Virology, Friedrich-Loeffler-Institut, Greifswald-Insel Riems, Germany; 6 Department of Neurosurgery, School of Medicine, Klinikum Rechts der Isar, Technical University of Munich, Munich, Germany; 7 Department of Neurology, University Hospital Agatharied, Agatharied, Germany; 8 Section of Clinical and Comparative Neuropathology, Centre for Clinical Veterinary Medicine, Ludwig-Maximilian University of Munich, Munich, Germany; 9 Department of Neurology, School of Medicine, Klinikum Rechts der Isar, Technical University of Munich, Munich, Germany; 10 Department of Neuroradiology, School of Medicine, Klinikum Rechts der Isar, Technical University of Munich, Munich, Germany

Even though the number of confirmed cases of bornavirus encephalitis (BE) rises constantly, the epidemiological risk of this fatal disease is still underrecognized. Human infection with Borna disease virus 1 (BoDV-1) was first proven in 2018 [3, 6] and the neuropathology of BE described in a systematic analysis of autopsies briefly thereafter [4].

Herein, we report on a BE patient exhibiting brain hemorrhage that was detected in magnet resonance imaging (MRI) and confirmed by histopathology post mortem. Moreover, we demonstrate that encephalic vessels can be infected by BoDV-1.

In December 2020, a 64-year-old otherwise healthy patient from Southern Germany developed flu-like symptoms. Ten days after first symptoms, progressive confusion and speech disorders resulted in hospitalization. Because of a clinically insignificant, subsegmental pulmonary embolism, mild anticoagulation with heparin was induced. Coagulation parameters were in the therapeutic range at all times. Despite treatment, the patient’s condition quickly deteriorated. MRI revealed restricted diffusivity as surrogate for cytotoxic changes and FLAIR hyperintensity as sign for edema predominantly in the basal nuclei, the insular and hippocampal region. Cerebrospinal fluid (CSF) analyses revealed an inflammatory syndrome and progressive blood–brain-barrier dysfunction. Because of inconspicuous microbiological und virologic workup (including negative CSF testing for BoDV-1 RNA), autoimmune encephalitis was assumed and treatment with high-dose corticosteroids and plasma exchange initiated. On day 26, a hemorrhagic transformation in the left insular region was seen in MRI (Fig. 1a, b).

**Fig. 1:**
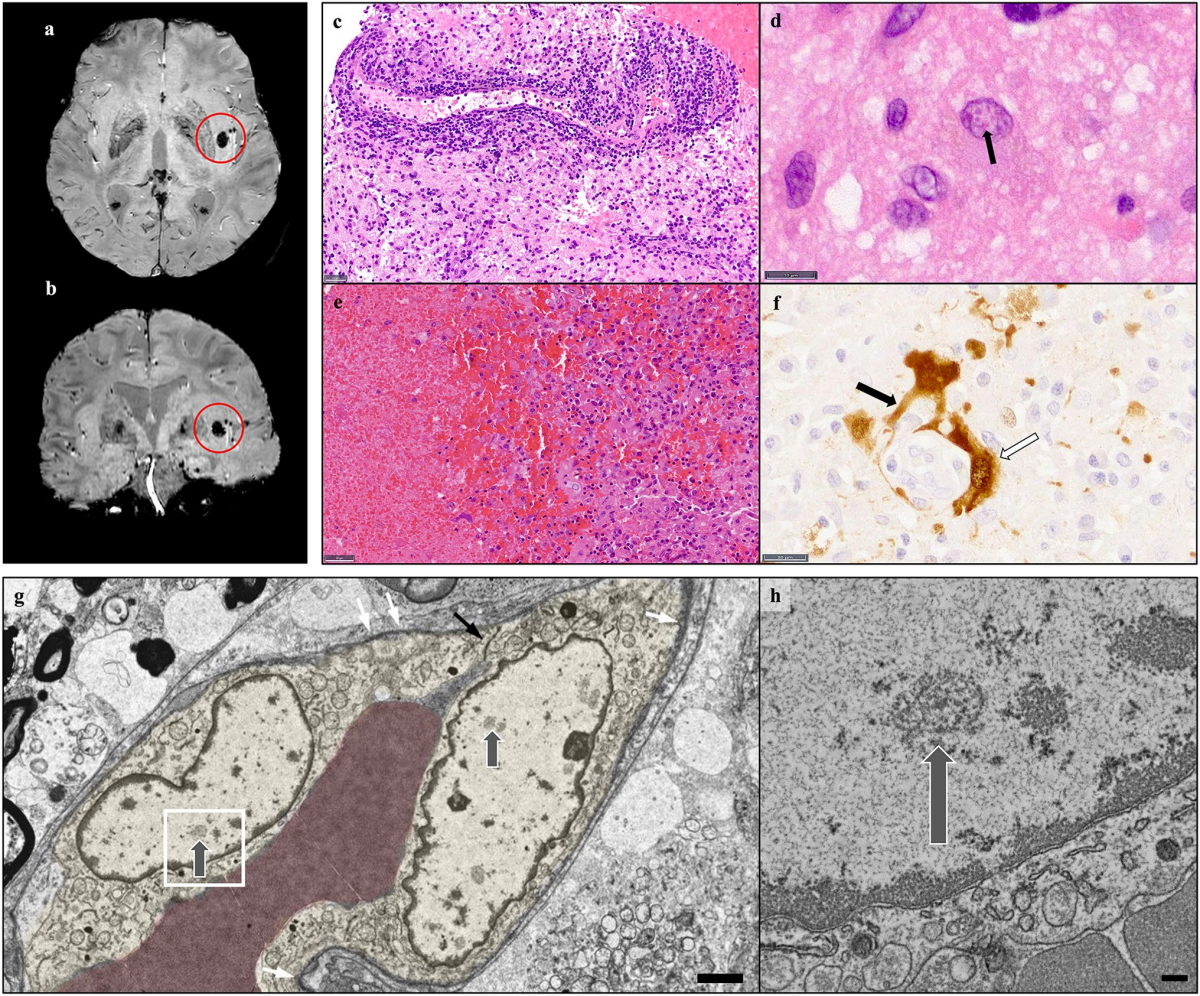
**a**,** b** MRI susceptibility weighted imaging (SWI) reveals a subinsular hemorrhage (red circles) 26 days after onset of symptoms. **c**, **d** Hematoxylin and eosin (HE) of a biopsy from the left caudate nucleus shows strong lymphocytic infiltrates (**c**, scale bar 20 μm) and astrocytes with eosinophilic intranuclear inclusion bodies (**d**, scale bar 10 μm). **e**, **f** In autoptic material, the hemorrhage with surrounding macrophages was seen (**e**, HE, scale bar 50 µm). Strikingly, a positive immunoreaction for BoDV-1 was detected in components of vessels (white arrow) and endfeet of astrocytes (black arrow) (**f**, scale bar 20 µm). **g** Electron microscopy shows two infected endothelial cells (yellow) with replication centers (grey arrows). For orientation, tight junctions (black arrow) and basal membranes (white arrows) are marked (scale bar 2 µm). **h** Higher magnification view of **g** (area within rectangle) displays detailed architecture of a viral replication center (arrow; scale bar 200 nm)

Due to lack of improvement, a brain biopsy from the caput of the left caudate nucleus was retrieved by neurosurgery on day 28. The biopsy revealed a pronounced lymphocytic infiltration (Fig. 1c) and strong glial and microglial activation. Eosinophilic intranuclear inclusion bodies of Cowdry type B were detected (Fig. 1d). Strikingly, prominent hemorrhagic changes were globally present. Due to typical histopathology (except for the hemorrhage), BoDV-1 immunohistochemistry was performed showing a positive precipitation. The definite diagnosis of BE was made on day 37. Presence of BoDV-1 RNA was confirmed by RT-qPCR as described earlier [5, 6]. Therapy with favipiravir was initiated on day 36, but did not result in any changes of the clinical condition. Progressive encephalitic changes involving the brainstem led to the patient’s death on day 43. The family gave informed consent to autopsy. Brain autopsy revealed a hemorrhage with maximal diameter of 1.2 cm in the left striatum, as described in MRI. The bleeding was surrounded by macrophages (Fig. 1e). Nearby vessels were highly infiltrated with lymphocytes. Strikingly, BoDV-1 immunohistochemistry showed a clear positive reaction in cell components of small vessels and end feet of astrocytes (Fig. 1f). This finding was corroborated by electron microscopy, which revealed viral replication centers in endothelial cells (Fig. 1g, h), and extravascular erythrocytes. Furthermore, our ultrastructural analysis revealed regions of demyelinated axons.

Otherwise, typical neuropathological changes of human BE that can be summarized as lymphocytic, sclerosing meningoencephalitis with microglial nodules and eosinophilic inclusion bodies [2] consistent with previous descriptions [1, 4] were also present.

This is the first report of a hemorrhagic lesion observed during BE in humans. Moreover, we proved virus infiltration of cell components of vessels by detection of BoDV-1 nucleoprotein and viral RNA replication centers, the latter clearly showing an endothelial involvement. This raises the question, if the observed hemorrhage is cause or consequence of this endothelial infection. Since BoDV-1 is a non-cytopathogenic virus [7], direct damage to the endothelium and bleeding as consequence would be contradictory to the virus’ nature. The infection of the vessels as consequence of the bleeding and pronounced hypoxic changes is a comprehensible pathomechanism. We hypothesize that the infection of the endothelium resulted from pre-damage of the vessels and might have spread from close infected astrocytes.

Even though this report is based on an individual case, it gives important new insights into the neuropathology of human BE.

## Abbreviations

BE: Bornavirus encephalitis
BoDV-1: Borna disease virus 1
CSF: Cerebrospinal fluid
H&E: Hematoxylin and eosin
MRI: Magnet resonance imaging
RT-qPCR: Reverse transcriptase quantitative polymerase chain reaction
SWI: Susceptibility weighted imaging
